# (3-Acetyl-4-methyl-1*H*-pyrazol-1-ide-5-carboxyl­ato)bis­(1,10-phenanthroline)nickel(II) 3.5-hydrate

**DOI:** 10.1107/S1600536813017194

**Published:** 2013-06-26

**Authors:** Sergey Malinkin, Anatoliy A. Kapshuk, Elzbieta Gumienna-Kontecka, Elena V. Prisyazhnaya, Turganbay S. Iskenderov

**Affiliations:** a Department of Chemistry, Kiev National Taras Shevchenko University, Volodymyrska Street 64, 01601 Kiev, Ukraine; b Faculty of Chemistry, University of Wrocław, F. Joliot-Curie Street 14, 50-383 Wrocław, Poland; c Department of Chemistry, Kyiv National University of Construction and Architecture, Povitroflotsky Avenue 31, 03680 Kiev, Ukraine

## Abstract

The title compound, [Ni(C_7_H_6_N_2_O_3_)(C_12_H_8_N_2_)_2_]·3.5H_2_O, crystallizes as a neutral mononuclear complex with 3.5 solvent water mol­ecules. One of the water mol­ecules lies on an inversion centre, so that its H atoms are disordered over two sites. The coordination environment of Ni^II^ has a slightly distorted octa­hedral geometry, which is formed by one O and five N atoms belonging to the *N*,*O*-chelating pyrazol-1-ide-5-carboxyl­ate and two *N*,*N′*-chelating phenanthroline mol­ecules. In the crystal, O—H⋯O, N—H⋯O and O—H⋯N hydrogen bonds involving the solvent water mol­ecules and pyrazole-5-carboxyl­ate ligands form layers parallel to the *ab* plane. These layers are linked further *via* weak π–π inter­actions between two adjacent phenanthroline mol­ecules, with centroid-to-centroid distances in the range 3.886 (2)–4.018 (1) Å, together with C—H⋯π contacts, forming a three-dimensional network.

## Related literature

The work presented here continues studies of complexes based on pyrazolate ligands with transition metals, see: Klingele *et al.* (2009 ▶); Malinkin *et al.* (2009 ▶, 2012*a* ▶,*b* ▶,*c* ▶); Ng *et al.* (2011 ▶); Penkova *et al.* (2008 ▶, 2009 ▶); Meyer & Pritzkow (2000 ▶); Bauer-Siebenlist *et al.* (2005 ▶); Świątek-Kozłowska *et al.* (2000 ▶). For related structures, see: Zhong *et al.* (2009 ▶); Zheng *et al.* (2009 ▶); Bouchene *et al.* (2013 ▶); Fang & Wang (2010 ▶); Fritsky *et al.* (2004 ▶, 2006 ▶); Kanderal *et al.* (2005 ▶); Moroz *et al.* (2010 ▶). For the starting material, see: Sachse *et al.* (2008 ▶).
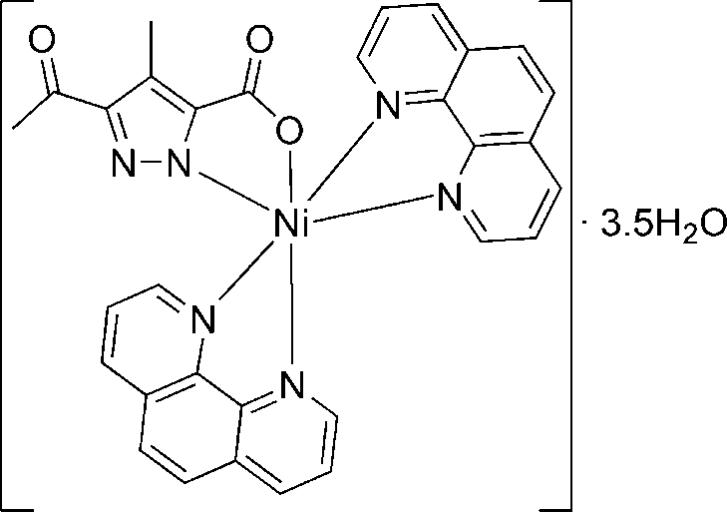


## Experimental

### 

#### Crystal data

[Ni(C_7_H_6_N_2_O_3_)(C_12_H_8_N_2_)_2_]·3.5H_2_O
*M* *_r_* = 648.29
Triclinic, 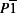

*a* = 9.865 (3) Å
*b* = 11.659 (4) Å
*c* = 13.561 (5) Å
α = 91.91 (3)°
β = 98.85 (3)°
γ = 105.20 (4)°
*V* = 1482.8 (9) Å^3^
*Z* = 2
Mo *K*α radiation
μ = 0.71 mm^−1^
*T* = 170 K
0.23 × 0.18 × 0.11 mm

#### Data collection

Nonius KappaCCD diffractometer
Absorption correction: numerical (*DENZO*/*SCALEPACK*; Otwinowski & Minor, 1997 ▶) *T*_min_ = 0.857, *T*_max_ = 0.929
12624 measured reflections
6830 independent reflections
3040 reflections with *I* > 2σ(*I*)
*R*_int_ = 0.070

#### Refinement

*R*[*F* ^2^ > 2σ(*F* ^2^)] = 0.062
*wR*(*F* ^2^) = 0.153
*S* = 0.85
6830 reflections
405 parameters
13 restraints
H-atom parameters constrained
Δρ_max_ = 1.12 e Å^−3^
Δρ_min_ = −0.59 e Å^−3^

### 

Data collection: *COLLECT* (Nonius, 2000 ▶); cell refinement: *DENZO*/*SCALEPACK* (Otwinowski & Minor, 1997 ▶); data reduction: *DENZO*/*SCALEPACK*; program(s) used to solve structure: *WinGX* (Farrugia, 2012 ▶); program(s) used to refine structure: *SHELXL97* (Sheldrick, 2008 ▶); molecular graphics: *ORTEP-3* (Farrugia, 2012 ▶) and *Mercury* (Macrae *et al.*, 2008 ▶); software used to prepare material for publication: *SHELXL97*.

## Supplementary Material

Crystal structure: contains datablock(s) I, global. DOI: 10.1107/S1600536813017194/sj5334sup1.cif

Structure factors: contains datablock(s) I. DOI: 10.1107/S1600536813017194/sj5334Isup2.hkl

Additional supplementary materials:  crystallographic information; 3D view; checkCIF report

## Figures and Tables

**Table 1:** Selected bond lengths (Å)

N1—Ni1	2.041 (4)
N3—Ni1	2.085 (4)
N4—Ni1	2.080 (4)
N5—Ni1	2.078 (3)
N6—Ni1	2.093 (4)
O2—Ni1	2.066 (3)

**Table 2:** Hydrogen-bond geometry (Å, °) *Cg* is the centroid of the N1/N2/C2/C3/C4 pyrazole ring.

*D*—H⋯*A*	*D*—H	H⋯*A*	*D*⋯*A*	*D*—H⋯*A*
O4—H4*A*⋯O3	0.95	2.31	3.088 (6)	139
O4—H4*B*⋯N2^i^	0.94	2.01	2.906 (6)	157
O5—H5*A*⋯O4^ii^	0.86	2.02	2.875 (6)	172
O5—H5*B*⋯O1	0.90	2.00	2.787 (5)	145
O6—H6*D*⋯O5	0.87	2.05	2.895 (6)	163
O6—H6*E*⋯O2	0.88	1.98	2.827 (5)	163
O7—H7*D*⋯O3	0.89	2.16	2.964 (4)	150
O7—H7*E*⋯O4	0.89	2.02	2.821 (5)	149
C12—H12⋯*Cg*1^iii^	0.93	2.77	3.646 (6)	158

Symmetry codes: (i) 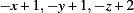; (ii) 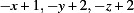; (iii) 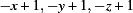.

**Fig. 1.**
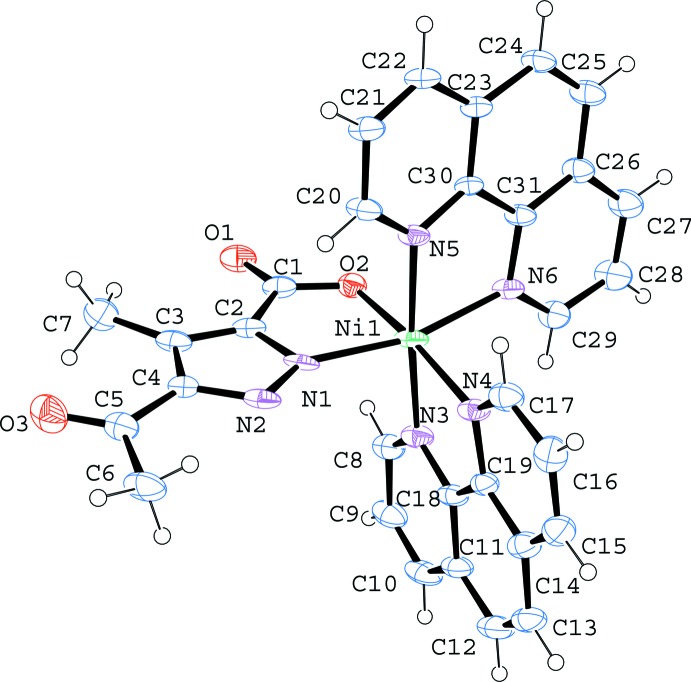
The molecular structure of (I), with displacement ellipsoids drawn at the 40% probability level.

**Fig. 2.**
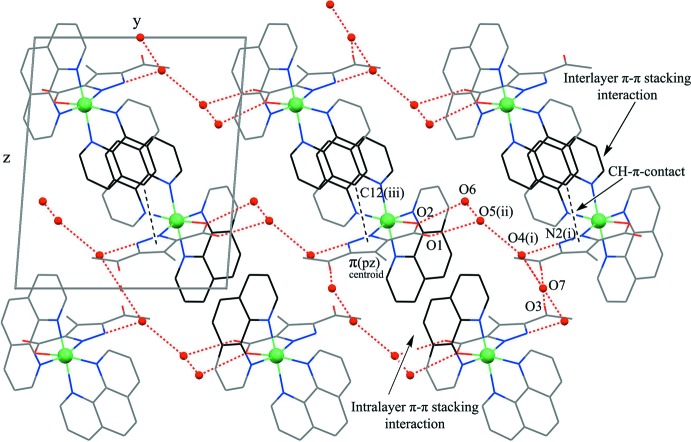
A portion of the packing, viewed down the y axis. Intermolecular hydrogen bonds link the molecules into a two-dimensional network. Hydrogen bonds and π-interactions are shown as red and black dashed lines, respectively. [Symmetry codes: (i) -x + 1, -y + 1, -z + 2; (ii) -x + 1, -y + 2, -z + 2; (iii) -x + 1, -y + 1, -z + 1.]

## supplementary crystallographic information

### Comment

The bridging nature of pyrazolate provides the possibility to bring metal ions into close proximity, which results in arrays with interesting magnetic and catalytic properties (Klingele *et al.*, 2009; Malinkin *et al.*, 2012*b*; Ng *et al.*, 2011). Therefore, research focused on pyrazolate complexes with higher nuclearity is of special interest in the field of supramolecular and bioinorganic chemistry (Penkova *et al.*, 2008, 2009; Meyer & Pritzkow, 2000; Bauer-Siebenlist *et al.*, 2005). Mononuclear pyrazolate-based complexes bearing non-coordinated donor groups can potentially be used as building blocks for the synthesis of discrete clusters as well as extended frameworks that offer a wide range of possible applications. On the other hand, phenanthroline is often used in the synthesis of discrete polynuclear complexes in order to prevent formation of coordination polymers by blocking a certain number of vacant sites in the coordination sphere of a metal ion (Fritsky *et al.*, 2004, 2006). Herein we report the synthesis and crystal structure of the title compound, (I), as a continuation of our earlier work devoted to complexes based on non-symmetrical pyrazole ligands (Penkova *et al.*, 2008; Malinkin *et al.*, 2012*a*,*b*), in particular, 3-acetyl-4-methyl-1*H*-pyrazole-5 carboxylic acid (Malinkin *et al.*, 2009, 2012*c*).

As shown in Figure 1, the Ni^II^ ion is coordinated by one pyrazolate ligand *via**N*,*O*-chelating groups and two *N*,*N*-chelating phenanthroline molecules forming a slightly distorted octahedral coordination environment. The Ni—N_pz_, Ni—N_phen_ and Ni—O distances are consistent with the reported data for related complexes (Fang & Wang, 2010; Zheng *et al.*, 2009; Zhong *et al.*, 2009; Bouchene *et al.*, 2013).

The coordinated pyrazolate ligand exhibits C—C, C—N, N—N bond lengths which are normal for bridging pyrazolate rings (Penkova *et al.*, 2008; Malinkin *et al.*, 2012*a*,*b*; Świątek-Kozłowska *et al.*, 2000). The C—O bond lengths in the deprotonated carboxylic groups differs significantly (1.239 (2) and 1.292 (2) Å) which is typical for monodentate coordinated carboxylates (Malinkin *et al.*, 2012*a*,*b*). Also the C—N and C—C bond lengths in the phenanthroline ligand are similar to those separations observed in other 2-substituted pyridine derivatives (Kanderal *et al.*, 2005; Moroz *et al.*, 2010).

In the crystal packing the complex molecules are associated *via* intermolecular hydrogen bonds (Table 1) that involve O—H and N—H interactions between the donor atoms of pyrazolate ligand and solvate water molecules forming layers which are parallel to the *xy* plane (Fig. 2). In addition layers are stabilized by a weak π–π interactions between phenanthroline moieties with intercentroid distances of 4.018 (1) Å. Further complex species are united into three-dimensional motif through a π–π interactions found between two adjacent phenanthroline molecules belonging to the different layers (intercentroid distances 3.886 (2) and 3.950 (2) Å) and a C—H(phenanthroline)···π(pyrazole) contacts (the shortest H—centroid separation is around 2.77 Å).

### Experimental

The compound was prepared by addition of 4 ml of a methanolic solution containing 0.0360 g (0.2 mmol) of phenanthroline and 0.0366 g (0.1 mmol) Ni(ClO_4_)_2_.6H_2_O to a mixture containing 0.0167 g (0.1 mmol) L (Sachse *et al.*, 2008) and 0.2 ml of aqueous NaOH solution (0.1 *M*) in 5 ml methanol.

Light-green crystals appeared after several days. Yield: 0.0227 g (35%). Elemental analysis calc. (%) for C_31_H_29_N_6Ni_O_6.5_: C 57.38; H 4.47; N 12.96; found: C 57.22; H 4.30; N 13.05.

### Refinement

The OH and NH hydrogen atoms were located from the difference Fourier map, and their positional and isotropic thermal parameters were included into the further stages of refinement. The C—H hydrogen atoms were positioned geometrically and were constrained to ride on their parent atoms, with C—H = 0.95–0.97 Å, and *U*_iso_ = 1.2–1.5 *U*_eq_(parent atom).

Large value of ratio *U*_eq_(max)/*U*_eq_(min) for O6 and O7 is caused by a slight disorder of the atoms. For this reason a command 'ISOR' was applied as a weak restraint.

### Crystal data

**Table:**

[Ni(C_7_H_6_N_2_O_3_)(C_12_H_8_N_2_)_2_]·3.5H_2_O	*Z* = 2
*M**_r_* = 648.29	*F*(000) = 674
Triclinic, *P*1	*D*_x_ = 1.452 Mg m^−^^3^
Hall symbol: -P 1	Mo *K*α radiation, λ = 0.71073 Å
*a* = 9.865 (3) Å	Cell parameters from 11133 reflections
*b* = 11.659 (4) Å	θ = 3.4–36.5°
*c* = 13.561 (5) Å	µ = 0.71 mm^−^^1^
α = 91.91 (3)°	*T* = 170 K
β = 98.85 (3)°	Block, light green
γ = 105.20 (4)°	0.23 × 0.18 × 0.11 mm
*V* = 1482.8 (9) Å^3^	

### Data collection

**Table:**

Nonius KappaCCD diffractometer	6830 independent reflections
Radiation source: fine-focus sealed tube	3040 reflections with *I* > 2σ(*I*)
Horizontally mounted graphite crystal monochromator	*R*_int_ = 0.070
Detector resolution: 9 pixels mm^-1^	θ_max_ = 28.6°, θ_min_ = 3.0°
φ scans and ω scans with κ offset	*h* = −12→12
Absorption correction: numerical (*DENZO*/*SCALEPACK*; Otwinowski & Minor, 1997)	*k* = −15→15
*T*_min_ = 0.857, *T*_max_ = 0.929	*l* = −18→18
12624 measured reflections	

### Refinement

**Table:**

Refinement on *F*^2^	Primary atom site location: structure-invariant direct methods
Least-squares matrix: full	Secondary atom site location: difference Fourier map
*R*[*F*^2^ > 2σ(*F*^2^)] = 0.062	Hydrogen site location: inferred from neighbouring sites
*wR*(*F*^2^) = 0.153	H-atom parameters constrained
*S* = 0.85	*w* = 1/[σ^2^(*F*_o_^2^) + (0.0675*P*)^2^] where *P* = (*F*_o_^2^ + 2*F*_c_^2^)/3
6830 reflections	(Δ/σ)_max_ = 0.001
405 parameters	Δρ_max_ = 1.12 e Å^−^^3^
13 restraints	Δρ_min_ = −0.59 e Å^−^^3^

### Special details

**Table:**

Geometry. All e.s.d.'s (except the e.s.d. in the dihedral angle between two l.s. planes) are estimated using the full covariance matrix. The cell e.s.d.'s are taken into account individually in the estimation of e.s.d.'s in distances, angles and torsion angles; correlations between e.s.d.'s in cell parameters are only used when they are defined by crystal symmetry. An approximate (isotropic) treatment of cell e.s.d.'s is used for estimating e.s.d.'s involving l.s. planes.
Refinement. Refinement of *F*^2^ against ALL reflections. The weighted *R*-factor *wR* and goodness of fit *S* are based on *F*^2^, conventional *R*-factors *R* are based on *F*, with *F* set to zero for negative *F*^2^. The threshold expression of *F*^2^ > σ(*F*^2^) is used only for calculating *R*-factors(gt) *etc*. and is not relevant to the choice of reflections for refinement. *R*-factors based on *F*^2^ are statistically about twice as large as those based on *F*, and *R*-factors based on ALL data will be even larger.

### Fractional atomic coordinates and isotropic or equivalent isotropic displacement parameters (Å^2^)

**Table:**

	*x*	*y*	*z*	*U*_iso_*/*U*_eq_	Occ. (<1)
N1	0.6503 (4)	0.6632 (3)	0.7897 (2)	0.0294 (9)	
N2	0.5838 (4)	0.5594 (3)	0.8208 (2)	0.0322 (10)	
N3	0.7221 (4)	0.6920 (3)	0.5843 (3)	0.0331 (10)	
N4	0.8913 (4)	0.5870 (3)	0.6988 (2)	0.0303 (9)	
N5	0.9485 (4)	0.7889 (3)	0.8732 (2)	0.0293 (9)	
N6	1.0125 (4)	0.8544 (3)	0.6950 (2)	0.0299 (9)	
O1	0.5691 (4)	0.9471 (3)	0.7919 (2)	0.0416 (9)	
O2	0.7478 (3)	0.8848 (3)	0.7411 (2)	0.0318 (8)	
O3	0.2663 (4)	0.4867 (3)	0.9303 (3)	0.0518 (10)	
Ni1	0.82850 (7)	0.73904 (5)	0.73104 (4)	0.0312 (2)	
C1	0.6312 (6)	0.8686 (4)	0.7797 (3)	0.0316 (12)	
C2	0.5759 (5)	0.7458 (4)	0.8088 (3)	0.0315 (11)	
C3	0.4603 (5)	0.6920 (4)	0.8544 (3)	0.0332 (12)	
C4	0.4690 (5)	0.5759 (4)	0.8588 (3)	0.0287 (11)	
C5	0.3683 (6)	0.4731 (4)	0.8924 (3)	0.0365 (13)	
C6	0.3883 (6)	0.3506 (4)	0.8784 (4)	0.0508 (15)	
H6A	0.4691	0.3442	0.9250	0.076*	
H6B	0.4038	0.3372	0.8113	0.076*	
H6C	0.3045	0.2922	0.8898	0.076*	
C7	0.3526 (5)	0.7476 (4)	0.8889 (4)	0.0431 (13)	
H7A	0.3621	0.7487	0.9605	0.065*	
H7B	0.2584	0.7018	0.8592	0.065*	
H7C	0.3686	0.8277	0.8691	0.065*	
C8	0.6363 (5)	0.7446 (4)	0.5314 (3)	0.0383 (13)	
H8	0.6268	0.8159	0.5584	0.046*	
C9	0.5574 (5)	0.6988 (5)	0.4354 (3)	0.0458 (14)	
H9	0.4957	0.7376	0.4009	0.055*	
C10	0.5754 (6)	0.5954 (5)	0.3951 (3)	0.0482 (16)	
H10	0.5254	0.5635	0.3321	0.058*	
C11	0.6679 (5)	0.5375 (4)	0.4475 (3)	0.0382 (13)	
C12	0.6942 (6)	0.4303 (5)	0.4122 (4)	0.0501 (16)	
H12	0.6486	0.3956	0.3489	0.060*	
C13	0.7818 (6)	0.3779 (4)	0.4666 (4)	0.0483 (15)	
H13	0.7965	0.3083	0.4404	0.058*	
C14	0.8553 (6)	0.4280 (4)	0.5668 (3)	0.0404 (14)	
C15	0.9472 (6)	0.3781 (4)	0.6280 (4)	0.0462 (14)	
H15	0.9663	0.3088	0.6052	0.055*	
C16	1.0106 (5)	0.4308 (4)	0.7228 (4)	0.0425 (13)	
H16	1.0728	0.3985	0.7644	0.051*	
C17	0.9775 (5)	0.5347 (4)	0.7536 (4)	0.0382 (13)	
H17	1.0194	0.5697	0.8174	0.046*	
C18	0.7407 (5)	0.5897 (4)	0.5443 (3)	0.0318 (12)	
C19	0.8312 (5)	0.5333 (4)	0.6038 (3)	0.0329 (12)	
C20	0.9158 (5)	0.7544 (4)	0.9607 (3)	0.0348 (12)	
H20	0.8343	0.6925	0.9614	0.042*	
C21	1.0000 (5)	0.8078 (4)	1.0528 (3)	0.0360 (12)	
H21	0.9734	0.7815	1.1128	0.043*	
C22	1.1195 (5)	0.8975 (4)	1.0534 (3)	0.0331 (12)	
H22	1.1747	0.9339	1.1138	0.040*	
C23	1.1596 (5)	0.9353 (4)	0.9623 (3)	0.0299 (11)	
C24	1.2904 (5)	1.0252 (4)	0.9547 (3)	0.0367 (12)	
H24	1.3502	1.0638	1.0128	0.044*	
C25	1.3267 (5)	1.0535 (4)	0.8642 (3)	0.0390 (13)	
H25	1.4124	1.1094	0.8612	0.047*	
C26	1.2346 (5)	0.9984 (4)	0.7734 (3)	0.0333 (12)	
C27	1.2673 (6)	1.0234 (4)	0.6772 (3)	0.0456 (14)	
H27	1.3521	1.0784	0.6704	0.055*	
C28	1.1733 (6)	0.9662 (4)	0.5937 (4)	0.0477 (15)	
H28	1.1940	0.9816	0.5300	0.057*	
C29	1.0464 (5)	0.8846 (4)	0.6058 (3)	0.0373 (12)	
H29	0.9819	0.8492	0.5487	0.045*	
C30	1.0704 (5)	0.8775 (3)	0.8739 (3)	0.0240 (10)	
C31	1.1071 (5)	0.9121 (4)	0.7786 (3)	0.0284 (11)	
O4	0.2548 (5)	0.6130 (4)	1.1314 (3)	0.0849 (15)	
H4A	0.3056	0.6009	1.0797	0.127*	
H4B	0.2955	0.5559	1.1627	0.127*	
O5	0.6423 (4)	1.1780 (3)	0.7310 (3)	0.0707 (13)	
H5A	0.6793	1.2428	0.7689	0.106*	
H5B	0.5979	1.1190	0.7654	0.106*	
O6	0.8621 (6)	1.0978 (3)	0.6534 (3)	0.1067 (19)	
H6D	0.8036	1.1361	0.6718	0.160*	
H6E	0.8406	1.0267	0.6755	0.160*	
O7	0.0000	0.5000	1.0000	0.383 (10)	
H7D	0.0556	0.4810	0.9600	0.575*	0.50
H7E	0.0563	0.5411	1.0543	0.575*	0.50

### Atomic displacement parameters (Å^2^)

**Table:**

	*U*^11^	*U*^22^	*U*^33^	*U*^12^	*U*^13^	*U*^23^
N1	0.040 (2)	0.0185 (19)	0.0159 (17)	−0.0093 (18)	−0.0078 (16)	−0.0005 (14)
N2	0.046 (3)	0.020 (2)	0.0188 (18)	−0.0036 (19)	−0.0079 (18)	0.0005 (15)
N3	0.043 (3)	0.023 (2)	0.027 (2)	−0.0004 (19)	0.0018 (19)	0.0016 (16)
N4	0.041 (3)	0.021 (2)	0.0210 (18)	−0.0037 (18)	0.0042 (17)	0.0008 (15)
N5	0.040 (3)	0.0186 (19)	0.0217 (18)	−0.0043 (17)	0.0037 (17)	−0.0003 (15)
N6	0.040 (2)	0.023 (2)	0.0198 (18)	0.0001 (18)	−0.0009 (17)	−0.0015 (15)
O1	0.054 (2)	0.0216 (18)	0.045 (2)	0.0063 (17)	0.0017 (17)	0.0011 (14)
O2	0.035 (2)	0.0276 (18)	0.0281 (17)	0.0016 (15)	0.0052 (15)	0.0003 (13)
O3	0.054 (3)	0.043 (2)	0.053 (2)	−0.0003 (19)	0.015 (2)	0.0153 (17)
Ni1	0.0433 (4)	0.0227 (3)	0.0173 (3)	−0.0043 (3)	−0.0027 (2)	−0.0026 (2)
C1	0.045 (3)	0.023 (3)	0.021 (2)	0.005 (2)	−0.004 (2)	0.0005 (19)
C2	0.042 (3)	0.023 (2)	0.023 (2)	0.003 (2)	−0.004 (2)	−0.0050 (18)
C3	0.043 (3)	0.025 (3)	0.022 (2)	0.001 (2)	−0.009 (2)	0.0006 (19)
C4	0.032 (3)	0.024 (2)	0.026 (2)	0.004 (2)	−0.002 (2)	−0.0038 (18)
C5	0.044 (3)	0.032 (3)	0.026 (2)	0.001 (2)	0.001 (2)	0.007 (2)
C6	0.068 (4)	0.026 (3)	0.044 (3)	−0.012 (3)	0.008 (3)	0.003 (2)
C7	0.048 (3)	0.029 (3)	0.051 (3)	0.007 (3)	0.008 (3)	0.007 (2)
C8	0.046 (3)	0.034 (3)	0.027 (2)	−0.004 (2)	0.007 (2)	0.003 (2)
C9	0.047 (3)	0.048 (3)	0.028 (2)	−0.007 (3)	−0.004 (2)	0.005 (2)
C10	0.053 (4)	0.053 (3)	0.018 (2)	−0.018 (3)	−0.002 (2)	0.001 (2)
C11	0.045 (3)	0.036 (3)	0.022 (2)	−0.010 (2)	0.007 (2)	−0.005 (2)
C12	0.060 (4)	0.043 (3)	0.031 (3)	−0.016 (3)	0.010 (3)	−0.012 (2)
C13	0.062 (4)	0.031 (3)	0.041 (3)	−0.014 (3)	0.023 (3)	−0.011 (2)
C14	0.054 (4)	0.029 (3)	0.032 (3)	−0.004 (3)	0.016 (3)	−0.002 (2)
C15	0.051 (4)	0.027 (3)	0.056 (3)	−0.004 (3)	0.025 (3)	−0.004 (2)
C16	0.046 (3)	0.033 (3)	0.051 (3)	0.011 (3)	0.014 (3)	0.009 (2)
C17	0.042 (3)	0.030 (3)	0.036 (3)	0.000 (2)	0.005 (2)	0.002 (2)
C18	0.040 (3)	0.025 (2)	0.022 (2)	−0.008 (2)	0.005 (2)	−0.0001 (19)
C19	0.039 (3)	0.025 (2)	0.028 (2)	−0.008 (2)	0.014 (2)	−0.0038 (19)
C20	0.041 (3)	0.030 (3)	0.023 (2)	−0.006 (2)	−0.003 (2)	0.0000 (19)
C21	0.047 (3)	0.029 (3)	0.024 (2)	0.000 (2)	0.001 (2)	−0.0022 (19)
C22	0.042 (3)	0.028 (3)	0.022 (2)	0.005 (2)	−0.006 (2)	−0.0061 (19)
C23	0.037 (3)	0.020 (2)	0.027 (2)	0.004 (2)	−0.001 (2)	−0.0056 (18)
C24	0.040 (3)	0.024 (2)	0.036 (3)	−0.005 (2)	−0.001 (2)	−0.006 (2)
C25	0.043 (3)	0.021 (2)	0.044 (3)	−0.003 (2)	0.002 (2)	−0.002 (2)
C26	0.041 (3)	0.023 (2)	0.030 (2)	−0.002 (2)	0.005 (2)	0.0035 (19)
C27	0.051 (4)	0.035 (3)	0.042 (3)	−0.006 (3)	0.010 (3)	0.004 (2)
C28	0.059 (4)	0.046 (3)	0.030 (3)	−0.002 (3)	0.010 (3)	0.010 (2)
C29	0.045 (3)	0.037 (3)	0.025 (2)	0.002 (2)	0.007 (2)	0.006 (2)
C30	0.031 (3)	0.015 (2)	0.023 (2)	0.0038 (19)	0.0004 (19)	−0.0037 (17)
C31	0.036 (3)	0.018 (2)	0.024 (2)	−0.001 (2)	0.001 (2)	0.0013 (17)
O4	0.101 (4)	0.065 (3)	0.118 (4)	0.042 (3)	0.065 (3)	0.054 (3)
O5	0.067 (3)	0.044 (2)	0.093 (3)	0.008 (2)	−0.002 (2)	0.030 (2)
O6	0.205 (6)	0.043 (3)	0.097 (3)	0.033 (3)	0.098 (4)	0.027 (2)
O7	0.51 (2)	0.397 (17)	0.303 (16)	0.246 (16)	0.044 (14)	0.047 (13)

### Geometric parameters (Å, º)

**Table:**

N1—N2	1.333 (4)	C12—H12	0.9300
N1—Ni1	2.041 (4)	C12—C13	1.333 (7)
N1—C2	1.394 (6)	C13—H13	0.9300
N2—C4	1.368 (6)	C13—C14	1.461 (6)
N3—Ni1	2.085 (4)	C14—C15	1.387 (7)
N3—C8	1.312 (6)	C14—C19	1.401 (6)
N3—C18	1.361 (5)	C15—H15	0.9300
N4—Ni1	2.080 (4)	C15—C16	1.386 (7)
N4—C17	1.324 (6)	C16—H16	0.9300
N4—C19	1.386 (5)	C16—C17	1.401 (6)
N5—Ni1	2.078 (3)	C17—H17	0.9300
N5—C20	1.324 (5)	C18—C19	1.415 (7)
N5—C30	1.363 (5)	C20—H20	0.9300
N6—Ni1	2.093 (4)	C20—C21	1.413 (5)
N6—C29	1.337 (5)	C21—H21	0.9300
N6—C31	1.379 (5)	C21—C22	1.355 (6)
O1—C1	1.247 (5)	C22—H22	0.9300
O2—Ni1	2.066 (3)	C22—C23	1.404 (6)
O2—C1	1.308 (6)	C23—C24	1.453 (6)
O3—C5	1.240 (6)	C23—C30	1.406 (5)
C1—C2	1.482 (6)	C24—H24	0.9300
C2—C3	1.395 (6)	C24—C25	1.358 (6)
C3—C4	1.382 (6)	C25—H25	0.9300
C3—C7	1.503 (7)	C25—C26	1.433 (6)
C4—C5	1.477 (6)	C26—C27	1.413 (6)
C5—C6	1.502 (7)	C26—C31	1.403 (6)
C6—H6A	0.9600	C27—H27	0.9300
C6—H6B	0.9600	C27—C28	1.374 (6)
C6—H6C	0.9600	C28—H28	0.9300
C7—H7A	0.9600	C28—C29	1.396 (6)
C7—H7B	0.9600	C29—H29	0.9300
C7—H7C	0.9600	C30—C31	1.438 (6)
C8—H8	0.9300	O4—H4A	0.9495
C8—C9	1.418 (6)	O4—H4B	0.9445
C9—H9	0.9300	O5—H5A	0.8599
C9—C10	1.371 (7)	O5—H5B	0.9001
C10—H10	0.9300	O6—H6D	0.8749
C10—C11	1.396 (7)	O6—H6E	0.8751
C11—C12	1.424 (7)	O7—H7D	0.8900
C11—C18	1.427 (5)	O7—H7E	0.8900
			
N2—N1—Ni1	140.1 (3)	C10—C11—C12	125.1 (5)
N2—N1—C2	107.9 (4)	C10—C11—C18	117.2 (5)
C2—N1—Ni1	111.9 (3)	C12—C11—C18	117.7 (5)
N1—N2—C4	107.4 (4)	C11—C12—H12	118.8
C8—N3—Ni1	127.8 (3)	C13—C12—C11	122.3 (5)
C8—N3—C18	118.8 (4)	C13—C12—H12	118.8
C18—N3—Ni1	113.2 (3)	C12—C13—H13	119.4
C17—N4—Ni1	130.8 (3)	C12—C13—C14	121.2 (5)
C17—N4—C19	116.1 (4)	C14—C13—H13	119.4
C19—N4—Ni1	113.1 (3)	C15—C14—C13	124.4 (5)
C20—N5—Ni1	128.9 (3)	C15—C14—C19	117.7 (4)
C20—N5—C30	117.7 (4)	C19—C14—C13	117.9 (5)
C30—N5—Ni1	113.1 (3)	C14—C15—H15	119.8
C29—N6—Ni1	130.3 (3)	C16—C15—C14	120.4 (5)
C29—N6—C31	117.0 (4)	C16—C15—H15	119.8
C31—N6—Ni1	112.6 (3)	C15—C16—H16	121.3
C1—O2—Ni1	116.3 (3)	C15—C16—C17	117.4 (5)
N1—Ni1—N3	92.73 (14)	C17—C16—H16	121.3
N1—Ni1—N4	99.54 (15)	N4—C17—C16	125.1 (4)
N1—Ni1—N5	91.36 (14)	N4—C17—H17	117.4
N1—Ni1—N6	165.31 (14)	C16—C17—H17	117.4
N1—Ni1—O2	80.47 (15)	N3—C18—C11	121.8 (5)
N3—Ni1—N6	96.35 (14)	N3—C18—C19	117.6 (4)
N4—Ni1—N3	79.56 (15)	C19—C18—C11	120.5 (4)
N4—Ni1—N6	93.46 (15)	N4—C19—C14	123.1 (5)
N5—Ni1—N3	175.80 (16)	N4—C19—C18	116.5 (4)
N5—Ni1—N4	98.84 (15)	C14—C19—C18	120.4 (4)
N5—Ni1—N6	79.83 (14)	N5—C20—H20	118.7
O2—Ni1—N3	91.62 (14)	N5—C20—C21	122.5 (4)
O2—Ni1—N4	171.18 (13)	C21—C20—H20	118.7
O2—Ni1—N5	89.97 (13)	C20—C21—H21	120.1
O2—Ni1—N6	87.73 (14)	C22—C21—C20	119.7 (4)
O1—C1—O2	124.6 (4)	C22—C21—H21	120.1
O1—C1—C2	121.4 (5)	C21—C22—H22	120.2
O2—C1—C2	114.0 (5)	C21—C22—C23	119.6 (4)
N1—C2—C1	117.3 (4)	C23—C22—H22	120.2
N1—C2—C3	109.9 (4)	C22—C23—C24	123.8 (4)
C3—C2—C1	132.8 (5)	C22—C23—C30	117.2 (4)
C2—C3—C7	128.1 (4)	C30—C23—C24	118.8 (4)
C4—C3—C2	102.8 (4)	C23—C24—H24	119.5
C4—C3—C7	129.1 (4)	C25—C24—C23	121.0 (4)
N2—C4—C3	112.0 (4)	C25—C24—H24	119.5
N2—C4—C5	119.7 (4)	C24—C25—H25	119.6
C3—C4—C5	128.2 (5)	C24—C25—C26	120.9 (4)
O3—C5—C4	120.8 (5)	C26—C25—H25	119.6
O3—C5—C6	119.8 (5)	C27—C26—C25	123.4 (4)
C4—C5—C6	119.4 (5)	C31—C26—C25	119.3 (4)
C5—C6—H6A	109.5	C31—C26—C27	117.3 (4)
C5—C6—H6B	109.5	C26—C27—H27	120.1
C5—C6—H6C	109.5	C28—C27—C26	119.7 (5)
H6A—C6—H6B	109.5	C28—C27—H27	120.1
H6A—C6—H6C	109.5	C27—C28—H28	120.5
H6B—C6—H6C	109.5	C27—C28—C29	119.1 (4)
C3—C7—H7A	109.5	C29—C28—H28	120.5
C3—C7—H7B	109.5	N6—C29—C28	123.7 (4)
C3—C7—H7C	109.5	N6—C29—H29	118.2
H7A—C7—H7B	109.5	C28—C29—H29	118.2
H7A—C7—H7C	109.5	N5—C30—C23	123.2 (4)
H7B—C7—H7C	109.5	N5—C30—C31	117.3 (3)
N3—C8—H8	118.2	C23—C30—C31	119.5 (4)
N3—C8—C9	123.6 (5)	N6—C31—C26	123.1 (4)
C9—C8—H8	118.2	N6—C31—C30	116.4 (4)
C8—C9—H9	121.2	C26—C31—C30	120.5 (4)
C10—C9—C8	117.7 (5)	H4A—O4—H4B	83.7
C10—C9—H9	121.2	H5A—O5—H5B	111.0
C9—C10—H10	119.6	H6D—O6—H6E	107.9
C9—C10—C11	120.8 (4)	H7D—O7—H7E	107.6
C11—C10—H10	119.6		

### Hydrogen-bond geometry (Å, º)

Cg is the centroid of the N1/N2/C2/C3/C4 pyrazole ring.

**Table:**

*D*—H···*A*	*D*—H	H···*A*	*D*···*A*	*D*—H···*A*
O4—H4*A*···O3	0.95	2.31	3.088 (6)	139
O4—H4*B*···N2^i^	0.94	2.01	2.906 (6)	157
O5—H5*A*···O4^ii^	0.86	2.02	2.875 (6)	172
O5—H5*B*···O1	0.90	2.00	2.787 (5)	145
O6—H6*D*···O5	0.87	2.05	2.895 (6)	163
O6—H6*E*···O2	0.88	1.98	2.827 (5)	163
O7—H7*D*···O3	0.89	2.16	2.964 (4)	150
O7—H7*E*···O4	0.89	2.02	2.821 (5)	149
C12—H12···*Cg*1^iii^	0.93	2.77	3.646 (6)	158

Symmetry codes: (i) −*x*+1, −*y*+1, −*z*+2; (ii) −*x*+1, −*y*+2, −*z*+2; (iii) −*x*+1, −*y*+1, −*z*+1.
